# Supplementary material for: The Dual-Targeted Fusion Inhibitor Clofazimine Binds to the S2 Segment of the SARS-CoV-2 Spike Protein
Source: Viruses. 2024 Apr 20;16(4):640. doi: 10.3390/v16040640 (PMC11054727; doi:10.3390/v16040640)
Supplement: Supplementary file 1 [file viruses-16-00640-s001.zip › Supporting_Information.pdf]

Supporting Information

The Dual-Targeted Fusion Inhibitor Clofazimine Binds to the S2 Segment of the SARS-CoV-2 Spike Protein.

Matthew R. Freidel,[1] Pratiti A. Vakhariya,[1]
Shalinder K. Sardarni,[1] and Roger S. Armen[1*]

[1]Department of Pharmaceutical Sciences, College of Pharmacy,
Thomas Jefferson University, 901 Walnut St. Suite 918,
Philadelphia, Pennsylvania, 19170.

*Corresponding author, E-mail: roger.armen@jefferson.edu

Figure S1. Consensus binding site for Clofazimine 2 on the Nsp13 Helicase. (A) Ribbon structure of Nsp13 colored rainbow from the N-terminus in blue to the C-terminus in red where several domains are labeled. (B) Shown is the orientation of the REC2A and REC1B domains in orientation to the binding site where the molecular surface of 2 is shown. (C) A zoom-in view of the binding site from the same orientation showing the residues forming the binding site. (D) A similar view showing the orientation the RNA binding sites modeled from other helicases in the background.

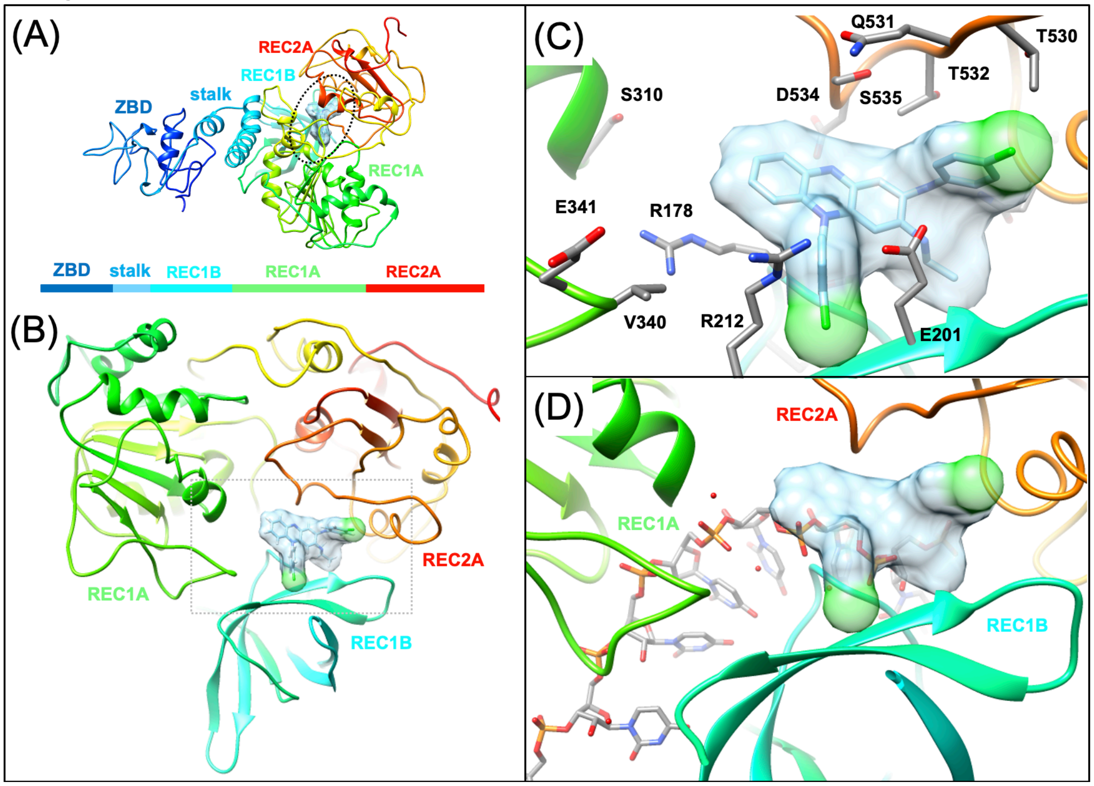

Table S1. Comparison of Clofazimine derivatives predicted binding free energy (ΔG_{bind}) at Site 1 and Site 2 to those approximated from experimental IC50 values. Predicted free energy of binding (ΔG_{bind}) in (kcal/mol) is calculated from a triplicate average (Avg) and standard deviation (Stdev) as described in methods.

	Compound<br>Deriv. ID	Observed<br>EC50<br>μM	Observed Calc.<br>ΔG<br>(kcal/mol)	Predicted Site 2<br>ΔG Avg<br>(kcal/mol)	Predicted Site 2<br>ΔG Stdev<br>(kcal/mol)	Predicted Site 1<br>ΔG Avg<br>(kcal/mol)	Predicted Site 1<br>ΔG Stdev<br>(kcal/mol)
1	**1**	10.0	-6.82	-9.06	0.16	-7.25	0.13
2	**6d**	154.0	-5.20	-8.71	0.10	-7.03	0.25
3	**6e**	16.1	-6.54	-9.79	0.48	-7.46	0.33
4	**7a**	141.0	-5.25	-8.70	0.08	-7.34	0.50
5	**7b**	39.3	-6.01	-8.60	0.08	-7.38	0.11
6	**7c**	40.9	-5.98	-8.69	0.19	-6.78	0.07
7	**7d**	14.5	-6.60	-9.14	0.22	-7.02	0.14
8	**7e**	13.5	-6.64	-9.00	0.07	-6.74	0.15
9	**7f**	10.3	-6.80	-8.95	0.17	-6.84	0.23
10	**7g**	16.6	-6.52	-9.56	0.14	-7.51	0.16
11	**7i**	10.0	-6.82	-9.47	0.23	-7.65	0.21
12	**7k**	12.4	-6.69	-10.0	0.23	-7.75	0.23
13	**7m**	17.2	-6.50	-8.61	0.04	-6.68	0.12
14	**7o**	13.5	-6.64	-9.32	0.01	-7.19	0.05
15	**15a**	29.6	-6.17	-8.66	0.29	-6.97	0.21
16	**15b**	81.1	-5.58	-8.75	0.17	-7.11	0.03
17	**15f**	14.6	-6.59	-8.89	0.09	-7.34	0.15
18	**15g**	10.0	-6.82	-8.89	0.22	-7.49	0.41
19	**15h**	33.1	-6.11	-8.25	0.13	-7.30	0.08
